# Supplementary material for: End Sequence Analysis Toolkit (ESAT) expands the extractable information from single-cell RNA-seq data
Source: Genome Res. 2016 Oct;26(10):1397–410. doi: 10.1101/gr.207902.116 (PMC5052061; doi:10.1101/gr.207902.116)
Supplement: Supplemental Material [file supp_gr.207902.116_Supp_Materials.tar.gz › ESAT-esat_v0.1/doc/html/classbroad_1_1core_1_1annotation_1_1_annotation_utils.html]

ESAT: broad.core.annotation.AnnotationUtils Class Reference


ESAT


- Main Page
- Related Pages
- Packages
- Classes
- Files

- Class List
- Class Index
- Class Hierarchy
- Class Members

All Classes Namespaces Files Functions Variables Enumerator Pages

- broad
- core
- annotation
- AnnotationUtils

Static Public Member Functions |
Static Public Attributes |
List of all members

broad.core.annotation.AnnotationUtils Class Reference

|  |  |
| --- | --- |
| Static Public Member Functions | |
| static void | main (String[] args) throws IOException, ParseException |
|  | |

|  |  |
| --- | --- |
| Static Public Attributes | |
| static final String | USAGE |
|  | |

## Member Function Documentation

|  |  |  |  |  |  |
| --- | --- | --- | --- | --- | --- |
| static void broad.core.annotation.AnnotationUtils.main | ( | String[] | *args* | ) | throws IOException, ParseException |

static

Jesse June 19, 2012

Jesse June 24, 2012

Here is the call graph for this function:

## Member Data Documentation

final String broad.core.annotation.AnnotationUtils.USAGE

static

**Initial value:**

= "Usage: AnnotationUtils TASK=<task\_num> <task\_args>\n" +

"\tTasks:\n" +

"\t\t1. Take the intersection two annotation sets: -set1 <File containing first set> -set2 <File containing second set> -minScore <to filter by minimum annotation score>" +

"\n\t\t\t [-set1format <[BED], GFF or generic> -set2format <[BED], GFF or generic> -outformat <desired output format BED, GFF or generic, default is se1format>" +

"\n\t2. Take the difference of two annotation sets (all annotations in set1 that do not overlap set2): -set1 <File containing first set> -set2 <File containing second set> -minScore <to filter by minimum annotation score>" +

"\n\t\t [-set1format <[BED], GFF or generic> -set2format <[BED], GFF or generic> -outformat <desired output format BED, GFF or generic, default is se1format>" +

"\n\t3. Print out all elements in one set (set1) that overlap another (set2): -set1 <File containing first set> -set2 <File containing second set> " +

"\n\t\t [-set1format <[BED], GFF or generic> -set2format <[BED], GFF or generic> -outformat <desired output format BED, GFF or generic, default is se1format>" +

"\n\t4. Merge annotation file -in <annotation file (dowa not support standard input input at this point)> -out <output file name or standard output by default> [-format <[BED], GFF or generic>]" +

"\n\t5. Reformat annotation file -in <annotation file (dowa not support standard input input at this point)> -out <output file name or standard output by default> [-informat <[BED], GFF or generic> -outformat <BED,[GFF],generic]" +

"\n\t6. Shuffle scores -in <Annotation file> -out <output file name or standard output by default> [-informat <[BED], GFF or generic>]" +

"\n\t7. Get overlap statistics of two sets (how much each of the elements in set1 is covered by set2). " +

"\n\t\t [-set1format <[BED], GFF or generic> -set2format <[BED], GFF or generic> -outformat <desired output format BED, GFF or generic, default is se1format>" +

"\n\t8. Take the complement of the the first annotation set in the second set (similar to 2 but elements incompletely overlapping will be broken into the regions that don't overlap): -set1 <File containing first set> -set2 <File containing second set> " +

"\n\t9. Filter any delimited file by extracting all records that overlap a given annotation file -in <Delimted file> -chrColumn <Column containing chrmosome information (first column is 0)> [-chr <Alternative if there is no chromosome information in the file, it is assumed that all positions are in a given chromosome>] -positionColumn <Column containing position information (first column is 0)> -annotations <Annotation file> -annotationFormat <[BED], GFF, Generic> [-sepeartor <Character separting columns, tab is assumed by default>]"+

"\n\t10. Print out all elements in one set (set1) that overlap another (set2) and are in the same orientation as the overlapping gene: -set1 <File containing first set> -set2 <File containing second set> " +

"\n\t\t [-set1format <[BED], GFF or generic> -set2format <[BED], GFF or generic> -outformat <desired output format BED, GFF or generic, default is se1format>" +

"\n\t11. Compute the probability that an interval of given size overlaps a given annotation set by chance: -set <File containing first set> -sizeFile <Size file with chromosome sizes> " +

"\n\t12. Print distance metrics: for each element in first set, print the distance to the closest element in the second set." +

"\n\t\t [-nonOverlapping <find the closest non-overlapping element> -set1format <[BED], GFF or generic> -set2format <[BED], GFF or generic> -chr <limit analysis to chr>" +

"\n\tSlideAndCount\tSlide a window over the annotation map and count elements that overlap each window" +

"\n\t\t -window <windowSize> -overlap <overlapSize> -chr <chr> -start <start> -end <end>" +

"\n\tOverlapSignifiance. Calculate the significance of the overlap between two sets of annotations by permuting across the same chromosome." +

"\n\t\t -set1 <set1> -set2 <set2> -nperm <number of permutations> -sizeFile <file of chromosome sizes>" +

"\n"

---

The documentation for this class was generated from the following file:

- java/broad/core/annotation/AnnotationUtils.java


---

Generated on Fri Aug 15 2014 15:10:50 for ESAT by  

 1.8.7
